# Sources of Variability in a Synthetic Gene Oscillator

**DOI:** 10.1371/journal.pcbi.1004674

**Published:** 2015-12-22

**Authors:** Alan Veliz-Cuba, Andrew J. Hirning, Adam A. Atanas, Faiza Hussain, Flavia Vancia, Krešimir Josić, Matthew R. Bennett

**Affiliations:** 1 Department of Biosciences, Rice University, Houston, Texas, United States of America; 2 Department of Mathematics, University of Houston, Houston, Texas, United States of America; 3 Department of Biology & Biochemistry, University of Houston, Houston, Texas, United States of America; 4 Department of Bioengineering, Rice University, Houston, Texas, United States of America; Stony Brook University, UNITED STATES

## Abstract

Synthetic gene oscillators are small, engineered genetic circuits that produce periodic variations in target protein expression. Like other gene circuits, synthetic gene oscillators are noisy and exhibit fluctuations in amplitude and period. Understanding the origins of such variability is key to building predictive models that can guide the rational design of synthetic circuits. Here, we developed a method for determining the impact of different sources of noise in genetic oscillators by measuring the variability in oscillation amplitude and correlations between sister cells. We first used a combination of microfluidic devices and time-lapse fluorescence microscopy to track oscillations in cell lineages across many generations. We found that oscillation amplitude exhibited high cell-to-cell variability, while sister cells remained strongly correlated for many minutes after cell division. To understand how such variability arises, we constructed a computational model that identified the impact of various noise sources across the lineage of an initial cell. When each source of noise was appropriately tuned the model reproduced the experimentally observed amplitude variability and correlations, and accurately predicted outcomes under novel experimental conditions. Our combination of computational modeling and time-lapse data analysis provides a general way to examine the sources of variability in dynamic gene circuits.

## Author Summary

A goal of synthetic biology is to design genetic circuits using mathematical models that predict circuit function. However, various sources of noise impact gene regulation in different ways. This hinders the development of accurate mathematical models, especially when single-cell accuracy is required. Here, we first experimentally characterize the noisy dynamics of a synthetic gene oscillator at the single-cell level. Then, using measurements obtained from the experiments, we develop a minimal computational model that correctly predicts the statistical behavior of single cells within a growing colony. Our method can be used to construct simple computational models that not only capture the average dynamics of gene circuits, but also the statistical properties of single cells.

## Introduction

Random fluctuations in gene networks have a variety of origins: *e.g*. small molecule numbers within cells [1–6], fluctuations in the environment [7, 8], spatial heterogeneity [9], or the cell cycle [10]. A number of experimental and theoretical studies have examined the impact of noise on gene networks in equilibrium. Frequently, such studies focus on decomposing fluctuations into an intrinsic component which affects individual genes independently, and an extrinsic component which impacts all reactions within the cell or population [3, 8, 11–15]. However, different sources of extrinsic and intrinsic noise can have distinct impacts on network dynamics. For instance, metabolic and environmental fluctuations can affect genetic oscillations differently than variability induced by cell division, although all three can be characterized as extrinsic noise sources. An approach that identifies the effect and origin of different fluctuation sources would allow us to develop more accurate computational models of genetic circuits, and better understand the processes that affect their function.

Here, we present a combined experimental and theoretical approach to identify the sources of noise in a synthetic dual-feedback oscillator [16]. We used microfluidic devices [17] to track the amplitude and period of oscillations in individual cells through multiple cell cycles. By following the entire lineage of progenitor cells, we were able to characterize fluctuations within and co-fluctuations between cells. We found that the period of oscillations varied little, while the amplitude varied considerably in time and across the population. Sister cells were highly correlated upon division, but these correlations decayed in time.

To explain the mechanism behind these observations, we introduced a series of increasingly detailed computational models. We started with a deterministic model to estimate parameters and then used a modified discrete stochastic simulation algorithm that accounted for transcriptional delay to describe fluctuations due to small molecule numbers [18–22]. However, this model was unable to reproduce the amplitude variability and the correlation between sister cells simultaneously. Only by including various other extrinsic noise sources was the model able to explain both the amplitude variability and correlation observed experimentally. By increasing model complexity in steps we obtained a minimal model that explained the data. Importantly, our model predicted the level of amplitude fluctuations and correlations in a set of experiments we did not use in fitting it.

Our analysis showed that different sources of noise must be taken into account in order to explain the observed temporal and cell-to-cell amplitude variability and correlation in our population of synthetic oscillators. This approach to building minimal predictive models is quite general. Gradually increasing complexity by adding physiologically plausible sources of noise leads to a simple model that can explain the data. Subsequent cross-validation on separate experiments ensures that the model was not overfit. We have thus elucidated sources and consequences of variability within genetic oscillators, and provided a framework for constructing predictive models of complex dynamical gene networks.

## Results

**Fig 1:**
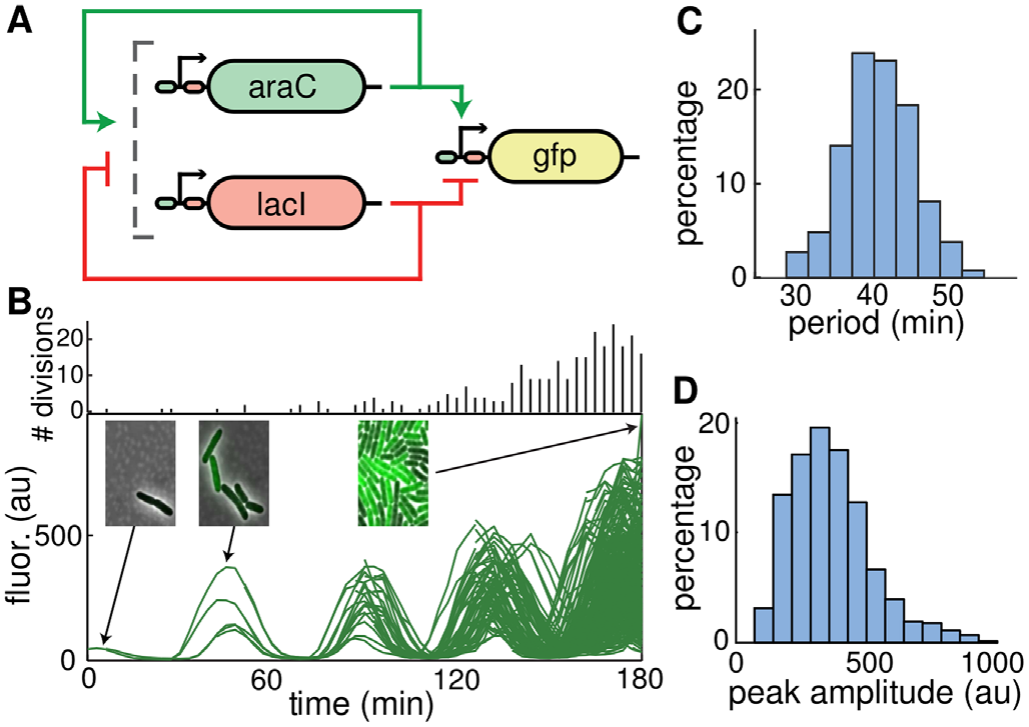
Variability in the synthetic dual-feedback oscillator. **A.** Circuit diagram of the oscillator. Transcription of *araC* and *lacI* is driven by identical copies of the P_*lac/ara-1*_ promoter, which is up-regulated by AraC in the presence of arabinose and repressed by LacI in the absence of isopropyl-*β*-D-1-thiogalactopyranoside (IPTG). **B.** Typical lineage fluorescence trajectory of the oscillator. Black bars at the top of the panel represent the number of cell divisions in each frame. Insets show representative images of some of the cells within the microfluidic trap (see Methods). **C.** Histogram of all recorded oscillation periods for traces shown in (B). The average period was 41 min, with coefficient of variation 0.11. **D.** Corresponding histogram of peak amplitudes. The mean peak amplitude was 317 (AU), with coefficient of variation 0.47.

We examined the dual-feedback oscillator described by Stricker *et al*. [16], which consists of three genes: two that encode the transcription factors AraC and LacI, and one that encodes the fluorescent reporter GFP (see Fig 1A). All three genes are under the control of the hybrid P_*lac/ara-1*_ promoter [23], which is up-regulated by AraC in the presence of arabinose and repressed by LacI in the absence of isopropyl-*β*-D-1-thiogalactopyranoside (IPTG). In addition, all three genes are tagged with the LAA version of the *ssrA* degradation sequence [24]. The presence of both the positive and negative feedback loops has been shown to support robust oscillations in the circuit [16, 25]. Since all genes are under the control of identical promoters, the concentration of GFP and the resulting fluorescence level provide a measure of the level of transcription in the oscillator.

### Amplitude variability and correlation in the oscillator

To measure the time-dependent GFP concentrations in individual cells, we used custom designed microfluidic devices that enable time-lapse fluorescence microscopy [26, 27]. We acquired phase contrast and fluorescence images every three minutes for three hours. We next segmented the images and tracked each cell and its fluorescence across time, keeping track of all lineages as cells grew and divided. At cell division, we kept track of each sister cell separately. Starting from a single cell, we thus obtained a branched trajectory: After the first division the trajectory split into two branches, and each successive division increased the number of branches by one. The resulting “lineage fluorescence trajectories” thus contained information from all descendants of the cell or cells initially placed in the trap, and about the relation between all descendants. Fig 1B shows the lineage trajectory for a single initial cell, illustrating the branching of trajectories at each cell division. Oscillations were maintained throughout the cell lineages.

Although all cells within a lineage are clonal copies of the initial cell, we observed large variability in oscillation amplitude (as measured by peak height) and smaller variability in oscillation period (Fig 1C–1D). Variability in period resulted in the divergence in the phase of the traces obtained from sister cell trajectories across the lineage. The average period of the entire population was 41 min, and variability in oscillation period (CV = 0.11) was small compared to the variability in amplitude (CV = 0.47). Computing the statistics for each lineage separately yielded similar results (see Methods).

**Fig 2:**
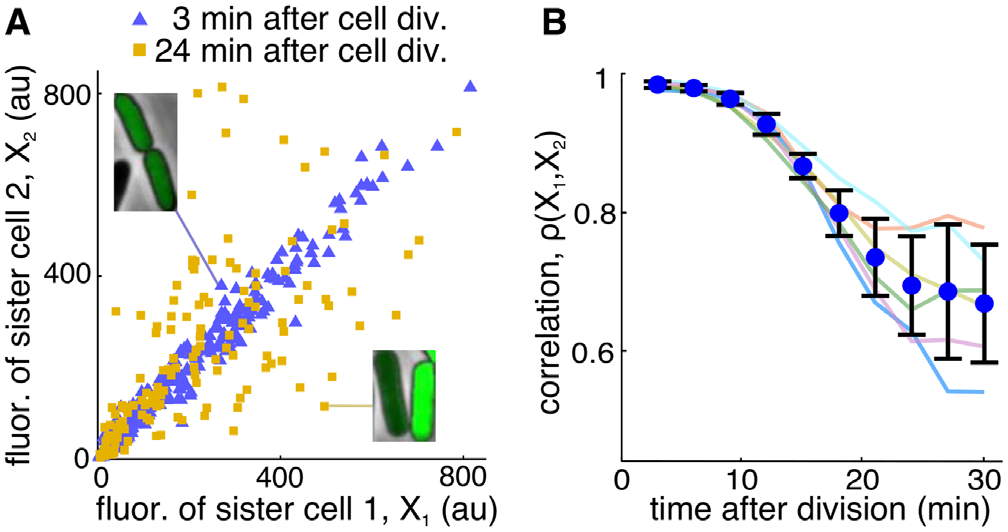
Correlation in fluorescence intensity of sister cells after cell division. **A.** Scatter plot of fluorescence intensity of sister cells *t* minutes after cell division (blue triangles, *t* = 3min; yellow squares, *t* = 24min). Three minutes after cell division, fluorescence intensity of sister cells is highly correlated with *ρX*_1_,*X*_2_ = 0.98. The correlation decreases to *ρX*_1_,*X*_2_ = 0.67 at 24 minutes after cell division. The insets show example pairs of sister cells. Data comes from approximately 150 pairs in the same lineage shown in Fig 1. **B.** Pearson correlation coefficient averaged across lineages as a function of time after cell division (blue circles, mean±sd; light colored curves, correlation functions for each of the 6 lineages).

To examine cell-to-cell co-variability in gene expression, we computed the Pearson correlation coefficient, *ρ*, of fluorescence between daughter cells *t* minutes after division using all pairs of daughter cells in a lineage (on average 175 pairs per lineage). In the first frame after division, fluorescence of two daughter cells was nearly identical (*ρX*_1_,*X*_2_(3) = 0.98, Fig 2A), indicating that protein partitioning was highly symmetric. This is consistent with previous results demonstrating that protein numbers after division follow a binomial distribution [28]. As time progressed, the correlation decreased as the trajectories of the daughter cells diverged. For instance, 24 min after cell division the correlation in fluorescence between sister cells decreased to *ρX*_1_,*X*_2_(24) = 0.67 (Fig 2B). Fig 2B also shows the correlation function of the 6 recorded lineages and their mean.

### Model of the dual-feedback oscillator: Intrinsic noise

To construct a mathematical model of the dual-feedback oscillator, we started with the following set of deterministic delay differential equations describing the dynamics of LacI (*r*), AraC (*a*), immature GFP (*g*), and mature GFP (*G*)

$$\begin{array}{ccc} \dot{r} & \;= & \;\alpha_{r}h\;\left[r_{\tau_{r}},a_{\tau_{r}}\right]-\frac{\gamma_{r}r}{R_{0}+r+a+g+G}-\beta r, \end{array}$$ (1)

$$\begin{array}{ccc} \dot{a} & \;= & \;\alpha_{a}h\;\left[r_{\tau_{a}},a_{\tau_{a}}\right]-\frac{\gamma_{a}a}{R_{0}+r+a+g+G}-\beta a, \end{array}$$ (2)

$$\begin{array}{ccc} \dot{g} & \;= & \;\alpha_{g}h\;\left[r_{\tau_{g}},a_{\tau_{g}}\right]-\frac{\gamma_{g}g}{R_{0}+r+a+g+G}-\lambda g-\beta g, \end{array}$$ (3)

$$\begin{array}{ccc} \dot{G} & \;= & \;\lambda g-\frac{\gamma_{G}G}{R_{0}+r+a+g+G}-\beta G,\; \end{array}$$ (4)

where *x*_*τ*_ = *x*(*t* − *τ*) for *x* in {*r*, *a*, *g*}, and

$$h\left(r,a\right)=\frac{f^{-1}+\frac{a}{C_{a}}}{\left(1+\frac{a}{C_{a}}\right){\left(1+\frac{r}{C_{r}}\right)}^{2}},$$ (5)

is the composite Hill function describing the activity of the hybrid promoters as a function of activator and repressor concentrations. Here *α*_*r*_, *α*_*a*_ and *α*_*g*_ are the maximal production rates; *τ*_*r*_, *τ*_*a*_, and *τ*_*g*_ are the transcriptional delay times; *γ*_*r*_, *γ*_*a*_, *γ*_*g*_, *γ*_*G*_, and *R*_0_ are the Michaelis-Menten parameters for ClpXP mediated proteolysis; *C*_*a*_, and *C*_*r*_ are the concentrations needed for half-maximal induction and repression. Subscripts refer to repressor (*r*), activator (*a*), and immature GFP (*g*). In addition, *f* is a unitless measure of the strength of the activation by *a* compared to basal production; *λ* is the maturation rate of GFP; and *β* is the dilution rate due to cell growth. We fit this deterministic model to experimental data to estimate the parameters. To do so we fit the shape of the solution as well as the period (see Methods).

In experiments the exact number of proteins within each cell is unknown. However, we can tune the protein number in the model by appropriately scaling the system in Eqs 1–4 without affecting the dynamics. More precisely, when we scaled each variable using the transformation *x*(*t*)→*x*(*t*)/Ω as well as appropriately changing the parameters (see details in Methods), the protein numbers were changed, but the dynamics of the corresponding deterministic system in Eqs 1–4 had the same form. The parameter Ω can be interpreted as unitless volume when other parameters are assumed to be fixed, or as a scaling parameter when the volume is fixed. Here we used the second interpretation.

**Fig 3:**
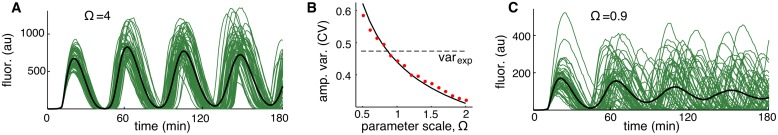
Stochastic single-cell simulations in the absence of extrinsic noise. **A.** Typical single-cell simulations with Ω = 4. Green curves represent the fluorescence intensity and the black curve is the average of 500 simulations (results of 50 simulations are shown). **B.** Dependence of variability in oscillation amplitude on the scaling parameter, Ω. Decreasing Ω increased the level of intrinsic noise and hence the variability in the amplitude of the oscillations (red circles). The experimentally observed amplitude variability (dashed line) is achieved when Ω = 0.9. The solid black curve corresponds to $0.44/\sqrt{\Omega }$, obtained by fitting a function proportional to $1/\sqrt{\Omega }$ to simulated data. **C.** Single-cell simulations with Ω = 0.9 show that, while amplitude variability matches that seen in experiments, the model period is much more variable (CV = 0.47).

To investigate the impact of noise due to finite size effects, we used a delayed stochastic simulation [18–21, 29] with rates obtained from the deterministic fit as propensity functions in the reactions of the associated birth-death process (see [22] and Methods). The parameter Ω directly controls the number of proteins in the system; for instance, doubling or tripling Ω, does the same to the number of proteins. Smaller values of the parameter Ω correspond to smaller numbers of proteins and hence larger intrinsic noise. For large values of Ω the model exhibits robust oscillations (*e.g*. Ω = 4 in Fig 3A), but amplitude variability is smaller than observed in experimental data. As expected, decreasing Ω led to increased amplitude variability (Fig 3B), matching the experimentally observed value when Ω = 0.9 (See Fig 3C). However, at this value of Ω the coefficient of variation (CV) of the oscillation period was 5 times higher than in experiments. Thus, a model that only included fluctuations due to small protein number could not account for the experimentally observer variability in amplitude and period.

### Lineage simulations and cell growth variability

**Fig 4:**
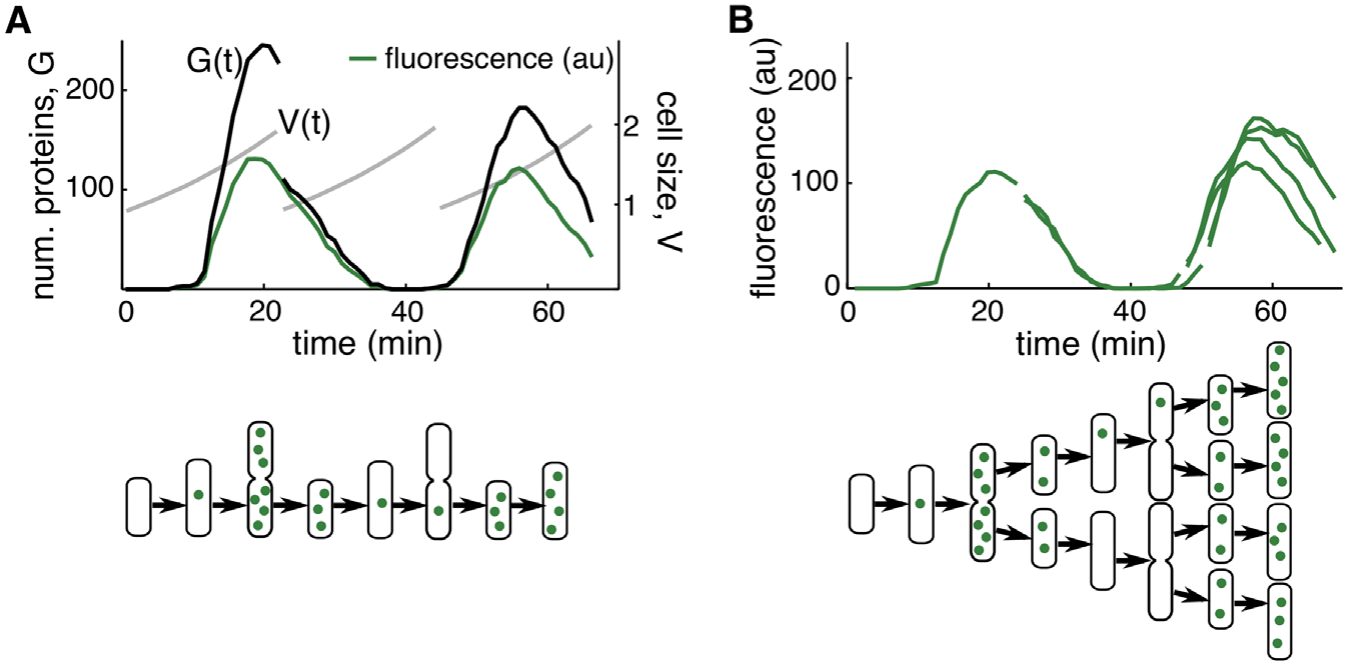
Incorporating the cell cycle into stochastic simulations. **A.** In a single cell simulation, a cell’s dynamics are modeled until it divides. After division the cell volume (*V*(*t*), gray) is halved and proteins (*G*(*t*), black) are partitioned binomially. Only one of the daughter cells is used in the remainder of the simulation. The concentration of *G* is *G*(*t*)/*V*(*t*) which results in a single cell trajectory (green). **B.** In a lineage simulation the volume of a cell and the number of proteins upon division are treated similarly to a single cell simulation. However, instead of throwing one daughter cell away at each division, the dynamics of both daughter cells are modeled and a lineage trajectory (green) is obtained.

We next asked if variability due to cell growth and division, and the associated random partitioning of proteins between daughter cells, could account for the discrepancies between our computational model and the experiments. To investigate this, we simulated the entire lineage of a progenitor cell (see Methods for details). In the stochastic version of Eqs 1–4 we accounted for cell growth only by incorporating dilution at rate *β*. Here, we instead accounted for cell growth explicitly by including an equation for cell volume, $\dot{V}=\beta V$, and scaling the production and proteolysis parameters to account for the doubling of molecular machinery such as plasmids and proteases (Fig 4A). As before, we simulated the system using the delayed stochastic simulation algorithm. When the volume of a cell reached the size at which division occurred, we replaced it with two daughter cells with half the volume, and partitioned all proteins (including those already created and the immature protein in the stack of delayed events) according to a binomial distribution [28]. We repeated these steps for each sister cell, thus simulating an entire lineage (Fig 4B). Furthermore, since we modeled cell growth explicitly, and cell growth and division exhibit variability [30], we allowed *β* to vary from cell to cell as well as the size required for division, *V*_max_. We estimated the distributions of *β* and *V*_max_ from experiments and used these estimates in simulations (see Methods). Thus, in addition to intrinsic noise, our lineage simulations included noise due to: 1) variability in growth rates between cells; 2) variability in size at division; and 3) protein partitioning at cell division.

**Fig 5:**
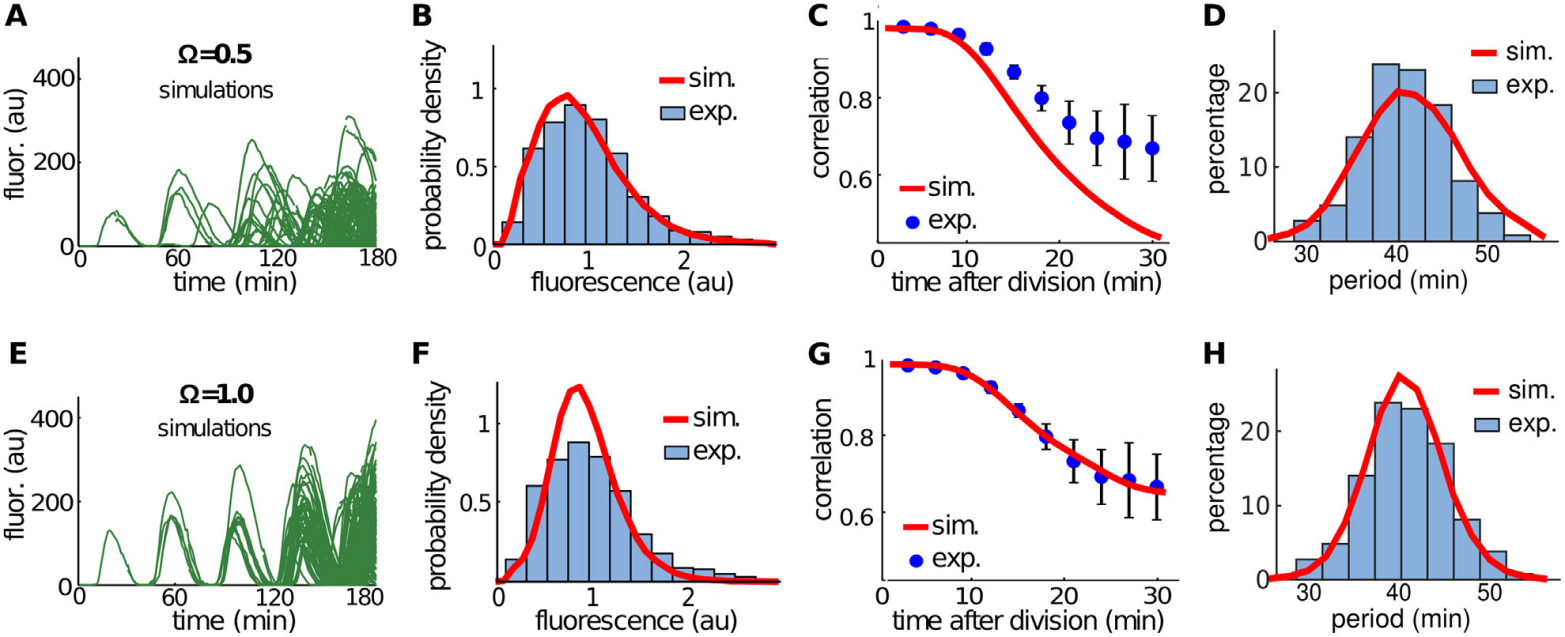
Lineage simulations in the absence of extrinsic noise. We chose the scaling parameter Ω to fit the amplitude variability (A-D, Ω = 0.5) or correlation between sister cells (E-H, Ω = 1.0) obtained from experimental data. **A, E.** Typical lineage simulations. **B, F.** Distribution of the oscillation amplitudes for experimental data (blue histograms) and from simulations (red curves), with amplitude rescaled by the mean. The coefficient of variation of the amplitude equals 0.47 for experiments, 0.47 for panel B, and 0.35 for panel F. **C,G.** Correlation in fluorescence intensity between sister cells after cell division for experimental data (blue circles, mean ± s.d.) and simulations (red curves). **D,H.** Distribution of the period for experimental data (blue histograms) and from simulations (red curves). The coefficient of variation of the period equals 0.11 for experiments, 0.14 for panel B, and 0.10 for panel F.

Fig 5A and 5D show typical simulations of lineage trajectories for two different values of Ω. The model matched experimentally observed amplitude variability most closely when Ω = 0.5 (Fig 5B). However, despite the close agreement in amplitude variability, lineage trajectories were qualitatively different from those observed experimentally (Fig 1B). We therefore compared correlations in fluorescence intensity between sister cells after cell division between simulations and experimental data. Simulations with Ω = 0.5 resulted in correlations that decayed faster than observed experimentally (Fig 5C). Decreasing intrinsic noise by setting Ω = 1.0 (Fig 5F), produced a good match. However, while the model oscillated robustly, intrinsic noise was now too small to match the experimentally observed amplitude variability (Fig 5E).

We therefore concluded that intrinsic noise and fluctuations in cell growth and protein numbers after cell division could not explain the experimentally observed variability. The level of intrinsic noise required to match the experimentally observed amplitude variability resulted in phase diffusion that was too fast, and correlations between sister cells that decayed too quickly. If we tried to match correlation decay, noise in our simulations was too small to reproduce the experimentally observed variability in amplitude.

### Parameter variability

Another source of variability are the random fluctuations in the cellular microenvironment and cellular resources necessary for gene expression [31–34]. This type of extrinsic noise can be viewed as variability in the parameters that describe protein creation. For instance, partitioning effects can result in fluctuations in protein numbers (as described in the previous section), but also increase variability in the molecular machinery responsible for protein production. If plasmids or enzymes needed for protein production are unevenly distributed between daughter cells upon division, transcription rates within the two daughter cells will differ. Parameter variability within gene circuits has been modeled previously in several ways. In the absence of cell division, Mondragón-Palomino *et al*. randomly sampled parameters for each realization of an oscillator model [35]. If cell division is explicitly modeled, however, parameters might change significantly only when a cell divides [36], or fluctuate continuously between divisions [14, 15].

We modeled parameter variability by sampling the value of parameters at each cell division. For a parameter subject to variability, for example *α*_*r*_, we sampled its new value after cell division from a distribution centered at the value of the parameter of the mother cell. The coefficient of variation of this distribution, Γ = *σ*_*α*_*r*__/〈*α*_*r*_〉, can then be used to tune parameter variability. Also, we incorporated a homeostatic mechanism in this distribution to ensure parameters did not diverge (see Methods for details). We focused on the variability of *α*_*r*_, *α*_*a*_, and *α*_*g*_ (Eqs (1–3)). These parameters were chosen because the activity of molecular machinery such as plasmid copy number, ribosomes, and energy in the cell, are expected to fluctuate more than parameters representing binding affinities, Hill coefficient, and degradation rates. All other parameters were fixed. We also explored alternative models of parameter variability, such as sampling of parameters independently of the values of parameters of the mother cell. We found that lineage dependence was necessary to be able to estimate Ω and Γ from experiments (see Methods).

**Fig 6:**
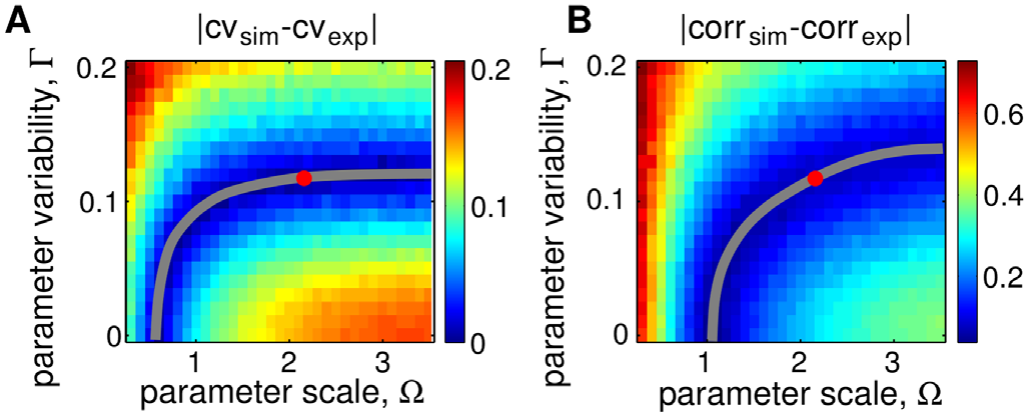
Heatmaps for the differences between amplitude variability and correlation in simulations and experiments as functions of parameter scale and variability. **A.** The difference between amplitude variability (gray curve shows contour of the minimum). **B.** The difference between the correlation function (gray curve shows the contour level that minimizes the difference). The intersection of the curves of minima shown in panels A and B, provides an estimate of the values of Ω and Γ. The two intersect at (Ω_0_, Γ_0_) ≈ (2.1, 0.12) (red dot in each panel).

In simulations we varied noise due to finite protein numbers by changing Ω, and parameter variability by changing Γ. We computed the coefficient of variation of the amplitude for a range of Ω and Γ and compared it to the coefficient of variation seen in experiments. Fig 6A shows the differences in the coefficient of variation of the amplitude between simulations and experiments, |CV_sim_ − CV_exp_|, as Ω and Γ were varied in the model. We also computed the correlation function from simulations, corr_sim_(*t*) (*t* = time after cell division), and compared it to the experimentally obtained correlation functions, corr_exp_(*t*). Fig 6B shows the differences in correlation between simulations and experiments, |corr_exp_ − corr_exp_| (using the *L* ^2^ norm), as Ω and Γ were varied in the model. As noted previously, in the absence of parameter variability, the experimentally observed amplitude variability can be matched in simulations with high intrinsic noise (small Ω). However, this same amplitude variability is observed over a range of Γ. Indeed, a decrease in intrinsic noise (increase in Ω) can be compensated by increasing parameter variability (Fig 6A, gray curve). Similarly, a good match for correlations in fluorescence can be obtained by appropriately tuning Ω and Γ (Fig 6B, gray curve). Matching concurrently the experimentally observed amplitude variability and correlation functions allows us to determine both Ω and Γ.

To explore further the dependence of amplitude variability and correlation on intrinsic and extrinsic noise, we used a simple oscillator model that captures the main features of the full model described above:

$$\begin{array}{cc} \text{d}r & =\rho \left(r_{0}-r\right)\text{d}t+\frac{1}{\sqrt{\Omega }}\text{d}\xi_{1}\;\\ \text{d}\theta & =\frac{2\pi }{T}\frac{r_{0}}{r}\text{d}t+\frac{1}{r\sqrt{\Omega }}\text{d}\xi_{2}, \end{array}$$ (6)

where *r* is the amplitude of a realization of the oscillation, *r*_0_ is the mean amplitude of the oscillations (in the absence of intrinsic noise), *ρ* determines the stability of the oscillator, *θ* is the phase, *T* is the period, d*ξ*_*i*_’s are independent standard white noise processes with zero mean and unit variance, and Ω controls the level of intrinsic noise (see Methods). Variability in *r*_0_ was modeled as in the full model using the coefficient of variation Γ as the parameter that controls extrinsic noise. We observed that intrinsic noise had a stronger effect on correlation than on amplitude variability; thus, large intrinsic noise can decorrelate cells faster as well as increase period variability. On the other hand, extrinsic noise has a stronger effect on amplitude variability. The same level of amplitude variability can be attained by decreasing intrinsic noise and increasing extrinsic noise (see Methods for details). By varying intrinsic and extrinsic noise it is thus possible to achieve large amplitude variability and high, persistent correlations between sister cells at the same time.

**Fig 7:**
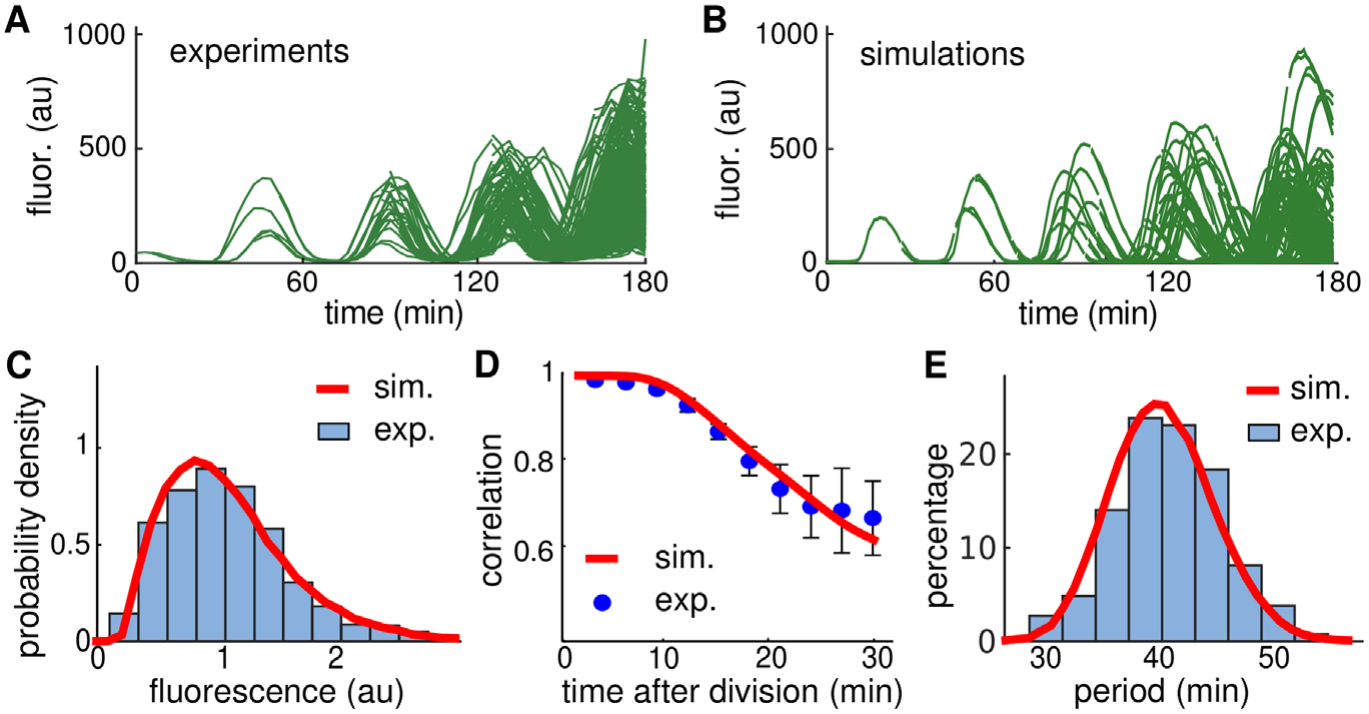
Comparison of experimental and simulated data with 2 mM IPTG. **A.** Experimental lineage trajectories obtained from time-lapse microscopy experiments. **B.** Simulated lineage trajectories of our model using the levels of intrinsic and extrinsic noise estimated in Fig 6 that match the experiments shown in A. **C.** Distribution of the oscillation amplitudes for experimental data shown in A (blue histograms) and from the simulations shown in B (red curves), with amplitude rescaled by the mean. **D.** Correlation in fluorescence intensity between sister cells after cell division for experimental data shown in A (blue circles, mean ± s.d.) and the simulations shown in B (red curves). **E.** Distribution of the period for experimental data shown in A (blue histograms) and from the simulations shown in B (red curves).

To validate our methodology experimentally, we compared the experimental lineage trajectories (Fig 7A) with simulated trajectories (Fig 7B) using the values of Ω and Γ estimated above. We observed that their qualitative behavior was similar; simulations and experiments exhibited robust oscillations with high amplitude variability. The distribution of the amplitudes and correlations in fluorescence show a close agreement between experiments and simulations (Fig 7C and 7D). Additionally, the coefficient of variation of the period, which we did not use to fit our model, was 0.10 in simulations (Fig 7E), close to the value observed in experiments (0.11).

**Fig 8:**
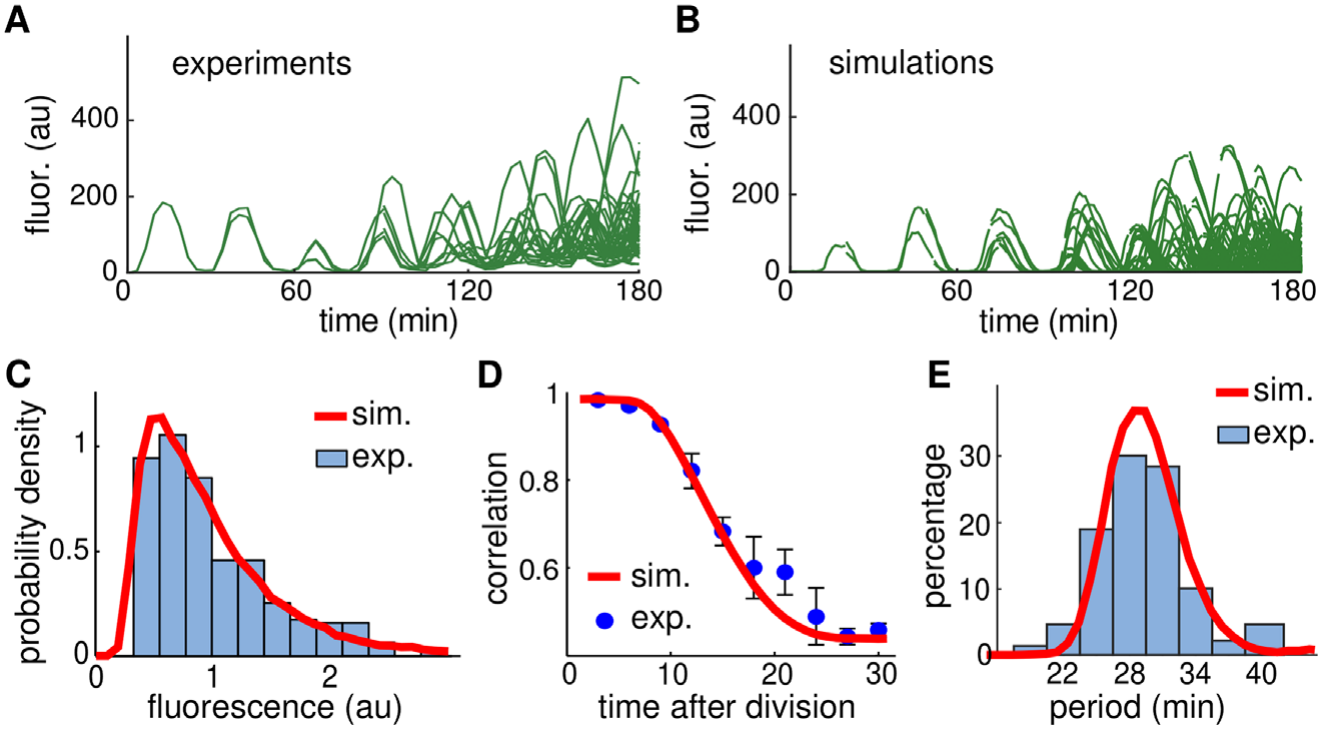
Cross-validating our model with 0 mM IPTG. **A.** Experimental lineage trajectories obtained from time-lapse microscopy experiments. **B.** Simulated lineage trajectories of our model using the levels of intrinsic and extrinsic noise estimated in Fig 6 that match the experiments shown in A. **C.** Distribution of the oscillation amplitudes for experimental data shown in A (blue histogram) and from the simulations shown in B (red curve), with amplitude rescaled by the mean. **D.** Correlation in fluorescence intensity between sister cells after cell division for experimental data shown in A (blue circles, mean ± s.d.) and the simulations shown in B (red curve). **E.** Distribution of the period for experimental data shown in A (blue histogram) and from the simulations shown in B (red curve).

To test the predictive power of our computational model we performed another experiment in which the concentration of IPTG was reduced from 2 mM to 0 mM. This reduction is known to decrease the period of the oscillator [16], and hence should change the amplitude variability and correlation between sister cells. The period at 0 mM IPTG was approximately 29 min (compared to ∼41 min in 2 mM IPTG), and amplitude variability and correlations were markedly different (Fig 8). To determine if our model could predict these changes, we decreased the parameter *C*_*r*_ in Eq (5) (corresponding to decreasing IPTG) until the model oscillated with the experimentally observed period. Importantly, only the parameter *C*_*r*_, which represents the amount of LacI needed for half-maximal repression of the promoter, was changed, while all other parameters were fixed at the values determined from our previous fit, including Ω and Γ. This allowed us to cross-validate our model using the experimentally observed variability and correlations at 0 mM IPTG. In experiments, amplitude variability increased, but the oscillations were still robust (Fig 8A); this was also observed in simulations (Fig 8B). The coefficient of variation of the amplitude was 0.55 in experiments, very close to the value predicted by simulations (0.56). We also compared the probability distribution of the amplitude in experiments and simulations, as well as the correlation functions (Fig 8C and 8D). The predicted distribution of amplitudes and the predicted correlation function matched the experimental data. Additionally, the coefficient of variation of the period was 0.19 in experiments (Fig 8E), close to the value predicted by simulations (0.17). These observations suggest that we did not overfit our model, and that our approach can be used to predict experimental outcomes. We also used the Kolmogorov-Smirnov test to compare the experimental and simulated amplitude distributions in Figs 7 and 8. In both cases they passed the KS test (within 95% confidence bound). The KS test also identified the two simulated distributions as different.

## Discussion

Understanding the sources and consequences of noise is key to designing robust synthetic genetic circuits. Oscillators provide an ideal platform for studying noise, as they fluctuate between large and small numbers of proteins. Hence, the relative impact of various noise sources changes with the oscillator’s phase. In experiments, we found that the amplitude exhibited high variability whereas the period did not, while GFP levels in sister cells were highly correlated for some time after cell division. Importantly, we determined that using just the amplitude variability to infer the level of intrinsic noise can result in model parameters inconsistent with other dynamical properties, such as correlation between sister cells after cell division. Instead, using a combination of intrinsic noise, cell cycle variability, and parameter variability we were able to obtain a model that better describes the stochastic dynamics of the oscillator. To estimate the impact of these sources of noise it was essential to track cell lineages across several generations. This allowed us to use long term correlations in addition to amplitude variability to distinguish between intrinsic noise and parameter variability.

We used parameter variability to capture multiple possible sources of noise which are not well understood. Other sources of variability that were not investigated were the genetic instability of synthetic circuits and the health of cells (filamentation, for example). However, these are very rare and difficult to examine due to the small number of occurrences, and unlikely to impact our overall conclusions due to the same reasons.

Since intrinsic noise has a fast time scale [28], it can quickly decorrelate the dynamics of sister cells. Other sources of noise can act on longer time scales [28]. This can result in large amplitude fluctuations from cycle to cycle, but a relatively slow decay of correlations between sister cells. Additionally, we found that it was necessary to implement long term correlations in parameter variability using a slow timescale. When our model did not include this feature, we could not find appropriate noise levels to explain amplitude variability and correlation at the same time (see Methods). Thus, if a gene network shows high amplitude variability but sister cells are correlated for an extended time after cell division, then extrinsic noise sources are likely to be the cause of such variability. We found that modeling the perturbation of parameters at cell division can capture these effects. Importantly, this type of perturbation covers sources of noise that affect protein synthesis, including unequal partitioning of cellular resources upon division or differences in cellular energy due to fluctuations in metabolic enzyme concentrations and local carbon source availability. Our method thus points towards a general strategy to identify the sources of noise in gene networks with complex dynamics.

We have examined the impact of different sources of noise in a system with relatively fast dynamics. These same sources could have a different impact in a slowly evolving system. However, our approach of using multiple statistical measures to characterize different aspects of the systems dynamics will work in such situations. For instance different sources of noise drive transitions between the two states of a genetic toggle switch [37, 38]. Variability in these transitions likely reflect the internal and external sources of fluctuations that drive the alternation between the states. Different sources of noise likely have a different effect on the statistics of transitions between states [39, 40]. Characterizing GFP variability, along with the first passage time distribution, and the decay of correlations in daughter cells could allow us to disentangle the different sources of fluctuations that contribute to this variability.

## Methods

### Microfluidic devices and microscopy

Devices were manufactured as in Hussain et al. (2014). Briefly, a 4” silicon wafer (Silicon Quest, San Jose, CA) was cleaned with acetone and isopropyl alcohol, and then dried with compressed nitrogen. The wafer was coated with SU-8 series photoresist (MicroChem, Newton, MA), then spun for 30 seconds in a spin coater (Brewer Instruments, Roala, MO) to distribute the resist. The wafer was baked at 95°C and let cool to room temperature. The wafer was mounted to the chuck of a mask aligner (SUSS, Germany). The photomask (CAD/Art Services, Bandon, OR) was mounted to the mask aligner, and the wafer was aligned to the photomask. The resist was exposed to UV light for cross-linking. The wafer was baked at 95°C to finalize cross-linking. Uncross-linked resist was removed with SU-8 developer (MicroChem, Newton, MA). The above steps were repeated until the device was completed. The wafer was hard-baked at 150°C to solidify the resist. To ensure PDMS liftoff, the wafer was coated with release agent (((tridecafluoro-1,1,2,2-tetrahydrooctyl)-1-trichlorosilane), Pfaltz & Bauer, Waterbury, CT) for 5 minutes under vacuum.

Polydimethylsiloxane (PDMS) polymer base and curing agent (Sylgaard 184, Dow Corning, Midland, MI) were mixed in a weigh boat at a 10:1 ratio until completely mixed (∼5 min). All bubbles were removed by degassing under vacuum. The mold for the selected device was wrapped in aluminum foil to contain the PDMS. Mixed PDMS was poured onto the wafer, and the degassing process was repeated. The wafer and PDMS were baked at 80°C for 2 hours. The cured PDMS monolith was removed from the wafer, and excess PDMS was trimmed from the monolith. Ports for fluidic connections were punched with a 0.5mm biopsy punch (GE Healthcare, Pittsburgh, PA). Individual chips were cut from the monolith. Chips were sonicated in methanol for 8 minutes. Methanol was poured off, and fresh methanol was added. Another round of sonication was performed for 8 minutes. Chips were baked at 80°C for 30 minutes to remove methanol. Chips were cleaned with tape (3M, St. Paul, MN). #1.5 coverslips (VWR, Radnor, PA) were cleaned with isopropyl alcohol and dried with compressed nitrogen. Chips and coverslips were cleaned in a UV/ozone oven (Jelight Co., Irvine, CA) for 3 minutes. Upon removal, chips were immediately inverted onto the coverslips to bind, and completed devices were baked at 80°C overnight to finalize binding.

**Fig 9:**
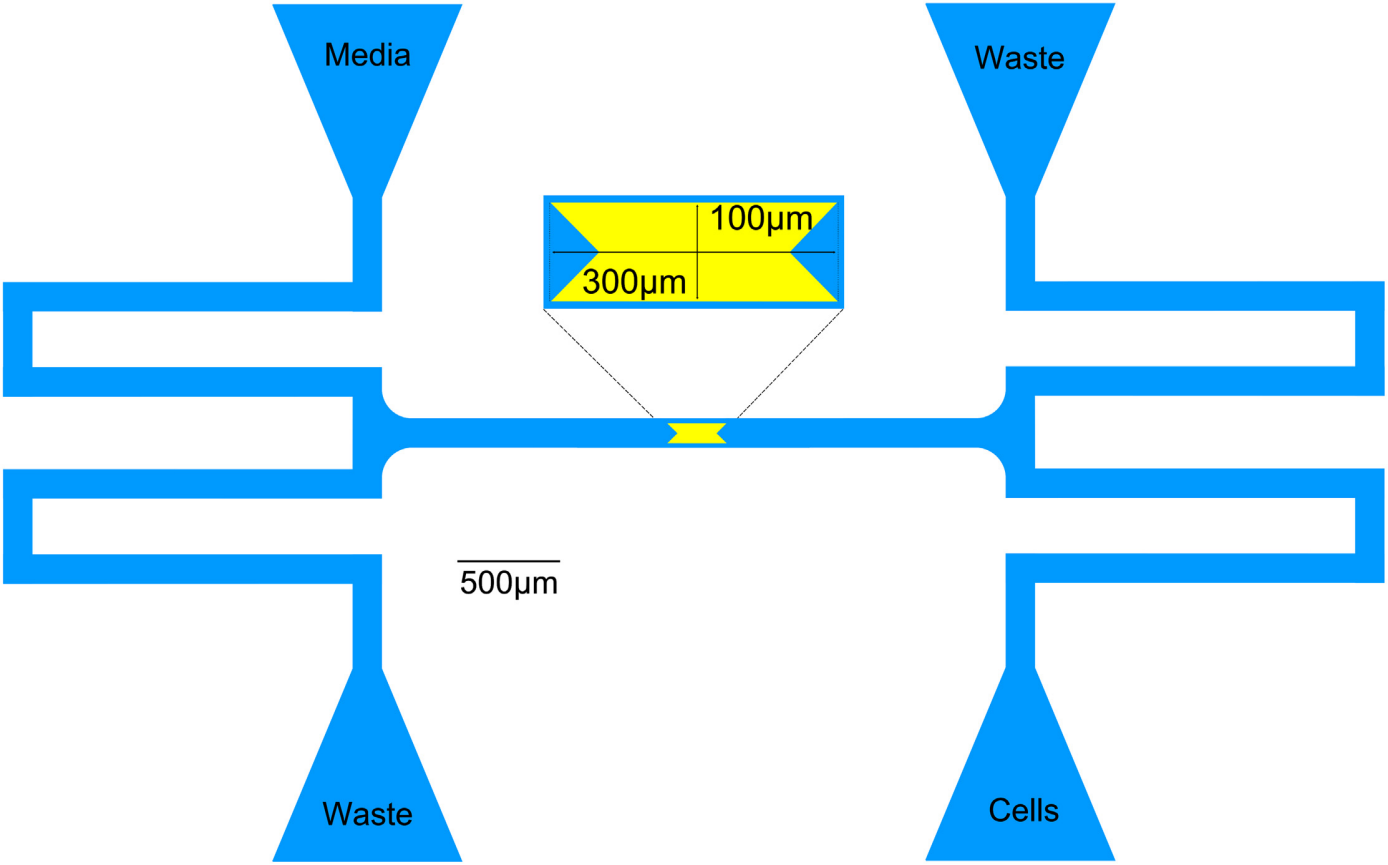
Schematics of microfluidic devices used in timelapse microscopy experiments.

Microscope experiments were prepared and performed as described in [27]. The activator (pJS167) and repressor (pZA14) plasmids (obtained from the Hasty lab, [16]) were transformed into Δ*araC*Δ*lacI* *E. coli* cells (JS006, [16]), and plated onto LB/agar plates with 100*μ*g/ml ampicillin and 50*μ*g/ml kanamycin. Plates were incubated overnight at 37°C and stored at 4°C. 5–10ml overnight cultures were inoculated from transformed cells, and incubated overnight at 37°C with shaking. Prior to experiments, overnight cultures were passed into 50ml fresh LB//100*μ*g/ml ampicillin//50*μ*g/ml kanamycin at a 1:1,000 dilution (*i.e*. 50*μ*l of overnight culture in 50ml of fresh media). Passed cultures were grown to an OD_600_ of 0.1–0.2, and 10ml was pelleted at 1,500×*g* for 5 minutes. Pelleted cells were resuspended in 10ml of fresh media with 100*μ*g/ml ampicillin and 50*μ*g/ml kanamycin prior to loading into the microfluidic device (Fig 9).

Microscopy experiments were conducted on a Ti-E inverted fluorescence microscope (Nikon Instruments Inc, Melville, NY) with an acrylic incubation chamber (In Vivo Scientific, St. Louis, MO). Microfluidic devices were prewarmed at 37°C for 30 minutes prior to flushing. Devices were flushed with 0.1% (v/v) Tween-20 (Sigma-Aldrich, St. Louis, MO). After flushing, syringes containing media (LB//100*μ*g/ml ampicillin//50*μ*g/ml kanamycin//2mM IPTG//0.7% (w/v) arabinose), resuspended cells, or sterilized water were connected to the microfluidic devices via 23 gauge luer stubs (Becton-Dickson, Franklin Lakes, NJ), Tygon microbore tubing (St. Gobain Performance Plastics, Paris, France) and 23-gauge pins (New England Small Tube Corp., Litchfield, NH). Cells were forced into the trapping area by flicking the line containing the resuspended cells. After trapping cells, the flow to the trapping area was adjusted to flow fresh media across the cells by changing the height of the syringe containing the resuspended cells. Trapped cells were imaged with a 100× objective every 3 minutes for 4–6 hours.

### Image analysis

**Fig 10:**
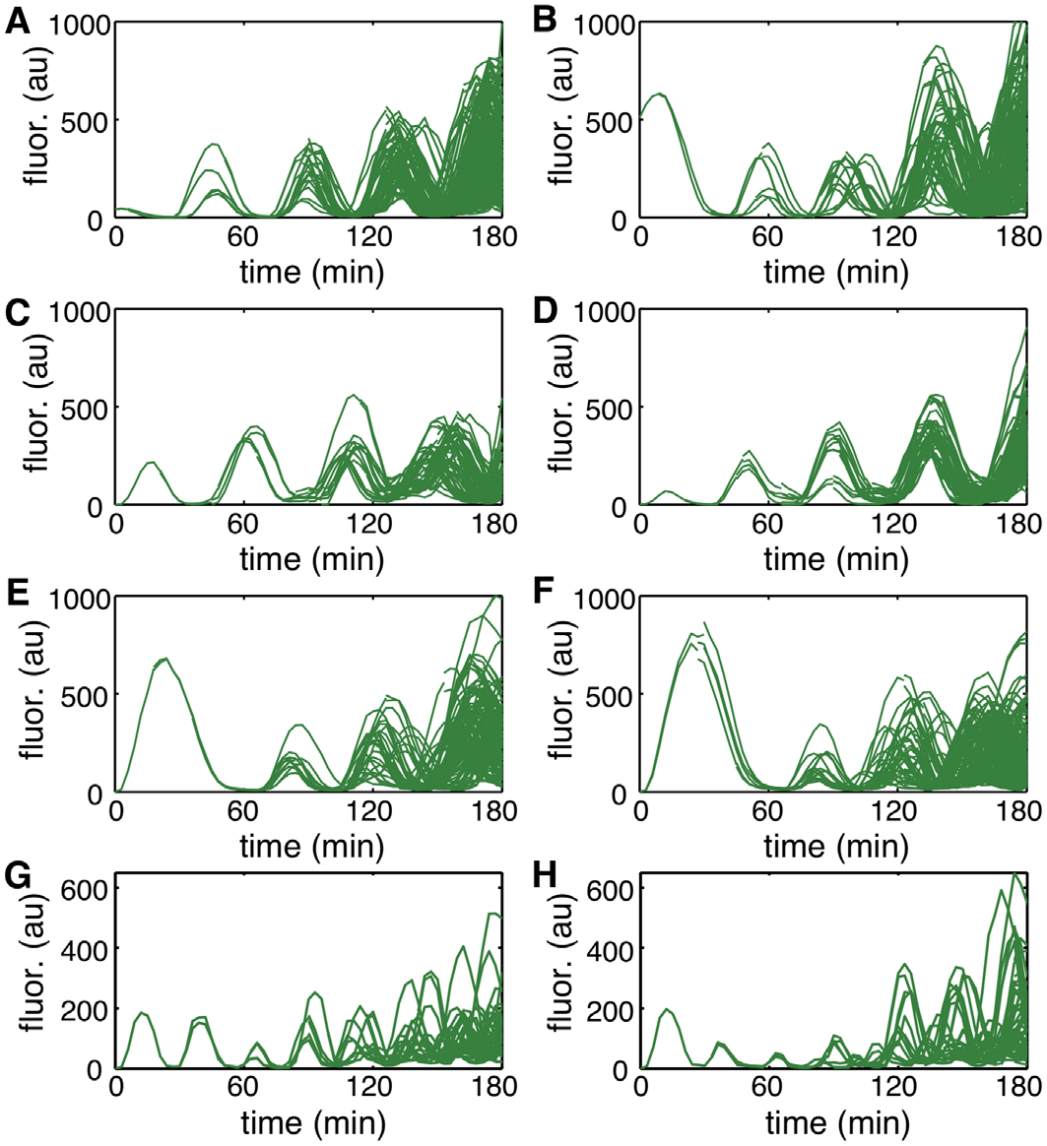
Lineages trajectories obtained from microscopy experiments using the cell-tracking algorithm. **A-F**. Lineage trajectories with 2mM IPTG. The coefficient of variation of the amplitude of all lineages combined is 0.47. The coefficient of variation within each lineage is 0.41, 0.47, 0.42, 0.33, 0.55, 0.56 (A-F, respectively), with an average value of 0.46. These 6 lineages were used for parameter estimation (Table 1, Fig 6), and for validation (Fig 7A–7D). **G-H**. Lineage trajectories with 0mM IPTG. The coefficient of variation of the amplitude of the two lineages combined is 0.55. The coefficient of variation within each lineage is 0.47 (G) and 0.66 (H), with an average value of 0.56. These two lineages were used for cross-validation only (Fig 7E–7H), and not for parameter estimation.

Segmentation of images was done manually. The tracking of cell lineage across images was done using our custom cell-tracking algorithm written in Matlab (available at github.com/alanavc/rodtracker): For each cell, *C*, in an image we found its position and length, *P*_*C*_ = (*x*, *y*) and *L*_*C*_, respectively. Then, we found all cells in the next image whose position *P*_*next*_ was near *P*, that is |*P*_*C*_ − *P*_*next*_| < *d*_*move*_. The parameter *d*_*move*_ equals the maximal movement of a cell from one frame to the next. From the cells satisfying this criterion, we selected cells with length *L*_*next*_ similar to *L*_*C*_, that is |*L*_*C*_ − *L*_*next*_| < *d*_*growth*_. The parameter *d*_*growth*_ equals the maximal expected growth between frames. We also found all pairs of cells whose length *L*_*next*,1_ and *L*_*next*,2_ approximately added up to *L*_*C*_, that is |*L*_*C*_ − (*L*_*next*,1_ + *L*_*next*,2_)| < *d*_*growth*_. With this criteria we created a “lineage graph” where each cell in an image had a set of possible transitions from one image to the next. Each transition corresponded to either movement between frames connecting the cell to a single descendant, or division and movement, connecting a cell to two descendants. This graph was then reduced by removing inconsistent transitions (e.g., a cell can only have one possible location in the next image or two locations if it divided). The reduced graph was further reduced by only selecting transitions that minimized ∑_*C*_(|*L*_*C*_ − *L*_*next*_| + |*P*_*C*_ − *P*_*next*_|). The final graph consisted of transitions where each cell is associated with a unique cell (if the cell moved) or two cells (if the cell moved and divided). The lineage trajectories were then computed using the lineage graph and the fluorescence data. One resulting lineage trajectory is shown in Fig 1B, and all trajectories are shown in Fig 10.

### Fitting parameters of the deterministic model to experimental data

To simplify parameter estimation of the model given in Eq (4) we followed these steps: Since all genes were under the control of identical promoters, and the copy number of the plasmids was approximately 60 for the activator and reporter, and 25 for the repressor [23], we considered *α*_*g*_ = 60*α* (monomer), *α*_*a*_ = 60*α*/2 (dimer), and *α*_*r*_ = 25*α*/4 (tetramer); where *α* is a parameter that controls the maximal production rate of proteins per plasmid when fully induced. Since the maturation rate does not change the total amount of GFP, but only the smoothness of the oscillations, it was not necessary to estimate this parameter from data. The half-time of maturation of fluorescence proteins ranges from 5 to 40min [41]. We set the maturation rate of GFP to *λ* = ln(2)/10min^−1^ (half-time equal to 10min), but as indicated before, our results do not depend on the precise value of *λ*. The promoter used in the dual-feedback oscillator is a strong promoter [23], and thus had a large maximal transcription rate, for which the delay affects the period but has little effect on its variability [42]. Thus, it was not necessary to estimate the delay parameters from data. We considered *τ*_*g*_ = 5min as the transcriptional delay for GFP production. Since araC is a dimer and lacI is a tetramer, we considered larger delays equal to *τ*_*a*_ = 5.5min and *τ*_*r*_ = 6.0min, respectively. The growth rate was estimated from phase-contrast microscopy to be approximately *β* = 0.0295min^−1^. We also assumed that the degradation of a mature GFP protein is identical to that of immature protein, and set *γ*_*G*_ = *γ*_*g*_. Thus, the set of parameters to be estimated is *P* = (*α*, *γ*_*r*_, *γ*_*a*_, *γ*_*g*_, *f*, *R*_0_, *C*_*a*_, *C*_*r*_).

To estimate the remaining parameters, we first needed to normalize the data from Fig 10A–10F. This was necessary, as the actual protein levels could not be determined exactly from our fluorescence measurements. First, we rescaled the trajectories so that oscillations with different amplitude are comparable: For the fluorescence trace obtained for each oscillation, *G*_*i*_(*t*), we found the peak time, *t*_*peak*_*i*__, and consider the trajectory 33 minutes before and after the peak. That is, we considered the time series *G*_*i*_(*t*) for *t*_*peak*_*i*__ − 33 ≤ *t* ≤ *t*_*peak*_*i*__ + 33. We chose 33 minutes because that includes the previous and next trough in the trajectory completely. We then centered the trajectories at *t* = 0; that is, we redefined *G*_*i*_(*t*): = *G*_*i*_(*t* + *t*_*peak*_) for −33 ≤ *t* ≤ 33 (here “≔” means reassignment, that is the quantity on the left side is assigned the value on the right side). We then normalized the time series using $G_{i}\left(t\right)≔\frac{G_{i}\left(t\right)-m_{i}}{M_{i}-m_{i}}$ where *m*_*i*_ = *min*{*G*_*i*_(*t*): −33 ≤ *t* ≤ 33} and *M*_*i*_ = *max*{*G*_*i*_(*t*): − 33 ≤ *t* ≤ 33}. This first normalization ensures that all peaks have height 1 and all troughs have height zero; thus, their magnitudes are now comparable. Finally, to obtain the “mean oscillation”, we took the average of all the shifted normalized oscillations: $G\left(t\right):=\frac{\sum_{i}G_{i}\left(t\right)}{\#\;\text{of oscillations}}$and normalized a second time to obtain the final time series

$$G\left(t\right)≔\frac{G(t)-min\{G(t):-33\leq t\leq 33\}}{max\{G(t):-33\leq t\leq 33\}-min\{G(t):-33\leq t\leq 33\}}.$$

**Fig 11:**
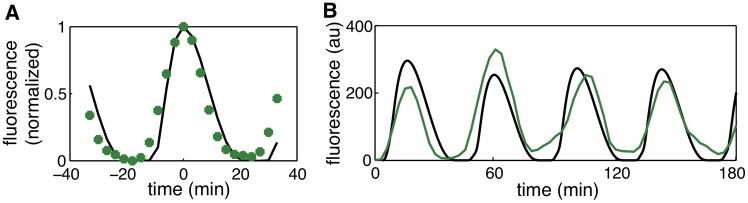
Fit of the model to the experimental data. **A.** Fit of the deterministic model (black) to the averaged and normalized experimental data (green, see text) using the estimated parameters from Table 1. **B.** Comparison of the fitted deterministic model to an experimentally measured single cell trajectory.

For each set of parameters *p*, we computed the error between simulations and experimental data as

$$E\left(p\right)=\sum_{-33\leq t\leq 33}\frac{{\left(G\left(t\right)-G_{sim}\left(t,p\right)\right)}^{2}}{G{\left(t\right)}^{2}}+{\left(\mu_{per}-sim_{per}\right)}^{2}/\mu_{per}^{2},$$

where *G*_*sim*_(*t*, *p*) is the data obtained from simulations using parameters *p* (centered at a peak), *μ*_*per*_ is the mean period of the experimental data, and *sim*_*per*_ is the period of the simulated data. The first term in *E*(*p*) measures the error in the shape of the oscillations and the second term measures the error in the period. We then used a gradient descend method to find the set of parameters, *p*, that minimized *E*(*p*). The value of the estimated parameters are given in Table 1. Fig 11 shows how the model fits experimental data.

**Table 1:** Parameters used in Eq (4). Parameters marked with * were estimated using experimental data shown in Fig 10A–10F.

**parameter**	**value**	**units**
α*	10.0	(molecules/cell) min^−1^
$\gamma_{r}^{*}$	8.0	(molecules/cell) min^−1^
$\gamma_{a}^{*}$	24.0	(molecules/cell) min^−1^
$\gamma_{g}^{*}$	48.0	(molecules/cell) min^−1^
$\gamma_{G}^{*}$	48.0	(molecules/cell) min^−1^
f*	17.0	1
$R_{0}^{*}$	0.0456	molecules/cell
$C_{a}^{*}$	0.791	molecules/cell
$C_{r}^{*}$	3.36	molecules/cell
*τ*_*g*_	5.0	min
*τ*_*a*_	5.5	min
*τ*_*r*_	6.0	min
*λ*	ln(2)/10	min^−1^
β*	0.0295	min^−1^

### Stochastic model and intrinsic noise

Based on the deterministic model in Eq (4), the stochastic model is given by the reactions

$$\begin{array}{cc} r\overset{\alpha_{r}h(r,a)}{\underset{\text{delayed}\;\tau_{r}}{\to }}r+1 & a\overset{\alpha_{a}h(r,a)}{\underset{\text{delayed}\;\tau_{a}}{\to }}a+1\\ g\overset{\alpha_{g}h(r,a)}{\underset{\text{delayed}\;\tau_{g}}{\to }}g+1 & r\overset{r(\beta +\gamma_{r}d(r,a,g,G))}{\to }r-1\\ a\overset{a(\beta +\gamma_{a}d(r,a,g,G))}{\to }a-1 & g\overset{g(\beta +\gamma_{g}d(r,a,g,G))}{\to }g-1\\ G\overset{G(\beta +\gamma_{G}d(r,a,g,G))}{\to }G-1 & g\overset{\lambda g}{\to }g-1,G+1 \end{array}$$ (7)

where

$$h\left(r,a\right)=\frac{f^{-1}+\frac{a}{C_{a}}}{\left(1+\frac{a}{C_{a}}\right){\left(1+\frac{r}{C_{r}}\right)}^{2}}\text{and}\;d\left(r,a,g,G\right)=\frac{1}{R_{0}+r+a+g+G}.$$

To simulate the system we used the delayed stochastic simulation algorithm (delayed SSA). This algorithm is an extension of the standard SSA [18], but for the delayed reactions the production of a protein is kept in a queue for the duration of the delay [19–21, 29].

In order to control the level of intrinsic noise in Eq (7), we first note that if we rescale all the variables in the deterministic model given in Eq (4) by the same factor, the scale of the system changes, but the dynamics do not. In other words, the levels of each protein can be rescaled so that the dynamics of the system is unchanged. In particular, using the rescaling *x* → *x*/Ω for each variable transforms the system of Eq (4) into

$$\begin{array}{cc} \dot{r} & =\Omega \alpha_{r}h_{\Omega }\left[r\left(t-\tau_{r}\right),a\left(t-\tau_{r}\right)\right]-\frac{\Omega \gamma_{r}r}{\Omega R_{0}+r+a+g+G}-\beta r,\\ \dot{a} & =\Omega \alpha_{a}h_{\Omega }\left[r\left(t-\tau_{a}\right),a\left(t-\tau_{a}\right)\right]-\frac{\Omega \gamma_{a}a}{\Omega R_{0}+r+a+g+G}-\beta a,\\ \dot{g} & =\Omega \alpha_{g}h_{\Omega }\left[r\left(t-\tau_{g}\right),a\left(t-\tau_{g}\right)\right]-\frac{\Omega \gamma_{g}g}{\Omega R_{0}+r+a+g+G}-\beta g-\lambda g,\\ \dot{G} & =\lambda g-\frac{\Omega \gamma_{G}G}{\Omega R_{0}+r+a+g+G}-\beta G, \end{array}$$ (8)

where

$$h_{\Omega }\left(r,a\right)=\frac{f^{-1}+\frac{a}{\Omega C_{a}}}{\left(1+\frac{a}{\Omega C_{a}}\right){\left(1+\frac{r}{\Omega C_{r}}\right)}^{2}}.$$

Thus, from the parameters in Table 1, we obtain a family of parameters

$$P_{\Omega }=\left(\Omega \alpha ,\Omega \gamma_{r},\Omega \gamma_{a},\Omega \gamma_{g},f,\Omega R_{0},\Omega C_{a},\Omega C_{r}\right),$$

for which the system in Eq (4) exhibits the same dynamics. Note that increasing Ω increases the number of each protein within the model. This is important, because we cannot infer the number of fluorescent proteins directly from the data. However, the amount of intrinsic noise within a biochemical system depends on the absolute number of the proteins within the circuit. Therefore, Ω controls the level of intrinsic noise in Eq (7)—increasing Ω *decreases* the level of intrinsic noise [43]. The parameter Ω can be interpreted as a unitless volume when parameters are fixed or as a scaling parameter when the volume is fixed. Here we used the second interpretation. We simulated 500 single-cell trajectories for each value of Ω in Fig 3B.

### Incorporating the cell cycle and lineage dependence into the stochastic model

To incorporate the cell cycle we account for cell growth explicitly and divide the volume of a cell by two when a cell divides. Thus, a variable for volume, *V*(*t*) (satisfying *V*′ = *βV*), was introduced in the model and the cell divides when *V*(*t*) = *V*_max_, where *V*_max_ is the volume required for division. At time *t* = 0, we start with a volume equal to 1, *V*_0_(0) = 1, and simulate the model until *V*_0_(*t*) = *V*_max_.

More precisely, if *y* is the state of the system and *x* is a protein of interest, then a reaction

$$x\overset{R(y)}{⟶}x+1$$

becomes

$$x\overset{VR(y/V)}{⟶}x+1$$

after incorporating cell size in the model [44]. The expression *y*/*V* is simply the concentration of proteins. Here *V* multiplies *R* because the molecular machinery in charge of production and degradation is being replicated proportionally to cell size (e.g. plasmids). This can also be justified starting from the deterministic model, incorporating cell size explicitly, and then incorporating stochasticity as follows. Consider a variable *x* that evolves according to

$$\frac{d}{dt}\left[x\right]=R\left(\left[y\right]\right)-\beta \left[x\right],$$

where [⋅] denotes concentration and *β* is the parameter corresponding to dilution due to cell growth. Since [*x*] = *x*/*V* and [*y*] = *y*/*V*, we obtain

$$\frac{d}{dt}\left(\frac{x}{V}\right)=R\left(\frac{y}{V}\right)-\beta \frac{x}{V}.$$

Since $\frac{d}{dt}(\frac{x}{V})=\left(x^{\prime }V-V^{\prime }x\right)/V^{2}$ and *V*′/*V* = *β*, we obtain

$$\dot{x}=VR\left(\frac{y}{V}\right),$$

which describes how *x* evolves incorporating volume explicitly. Finally, the stochastic implementation takes the form $x\to^{VR(y/V)}x+1$.

At cell division we divide the volume by half and partition all the existing proteins randomly into two groups corresponding to the two daughters using binomial partitioning. We similarly partition the stack of immature proteins in the production queue. We used a binomial distribution for partitioning (each cell is equally likely to receive a protein) [28]. Modeling cell division explicitly not only allows us to model variability caused by cell partitioning (Fig 4A), but it also allows us to simulate lineages (Fig 4B) and compare them directly to lineage trajectories from experimental data.

**Fig 12:**
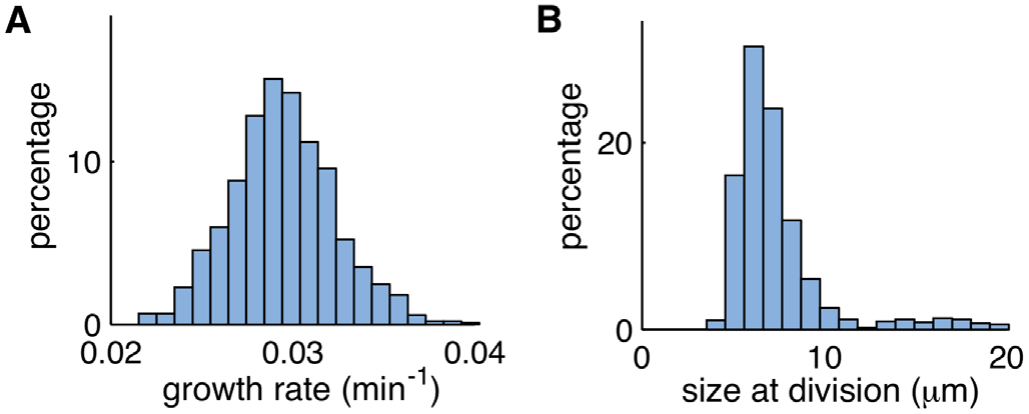
Histograms for growth rate and size required for division of the cell population. **A.** The growth rate was 0.0295min^−1^ on average and had a coefficient of variation of 0.099. **B.** The size of the mother cell at division was 7.7*μ*m on average with a coefficient of variation of 0.45.

Modeling cell division explicitly also allows us to incorporate the variability in growth rate (*β*) and the size required for division (*V*_max_). We estimated the variability from cell to cell of *β* and *V*_max_ (Fig 12), and incorporated this information in the simulations. The statistics of *V*_max_ and *β* were found from phase-contrast microscopy data. We used a normal distribution for *β* and a shifted-gamma distribution for *V*_max_: *β* = *β*_0_(1 + Γ_*β*_ *η*_1_) and *V*_max_ = *V*_base_ + *η*_2_, where $\eta_{1}∼N\left(0,1\right)$ and *η*_2_ ∼ *Gamma*(*k*, *θ*), where *k* and *θ* are the shape and scale of the gamma distribution (Table 2). We simulated 1000 lineages (8 generations each) for each value of Ω in Fig 5.

**Table 2:** Cell cycle parameters estimated from Fig 12.

**parameter**	**value**	**units**
Γ_*β*_	0.099	1
*V*_base_	4.5	*μm*
*k*	2.5	1
*θ*	1	1

We did not include variability in the size ratio of sister cells at division because it was not clear if the differences between sister cells sizes were due to real differences or due to errors in the segmentation of phase-contrast images. We estimated the variability of the size ratio (size of daughter cell/size of mother cell) to have a CV of about 0.09. The true variability between sister cells in cell size would be smaller. We checked the impact of including this overestimated value of size ratio variability in simulations used to obtain Fig 5, and the variability reported for Ω = 0.5, 1.0 changed by only 3%.

### Rate parameter variability

**Fig 13:**
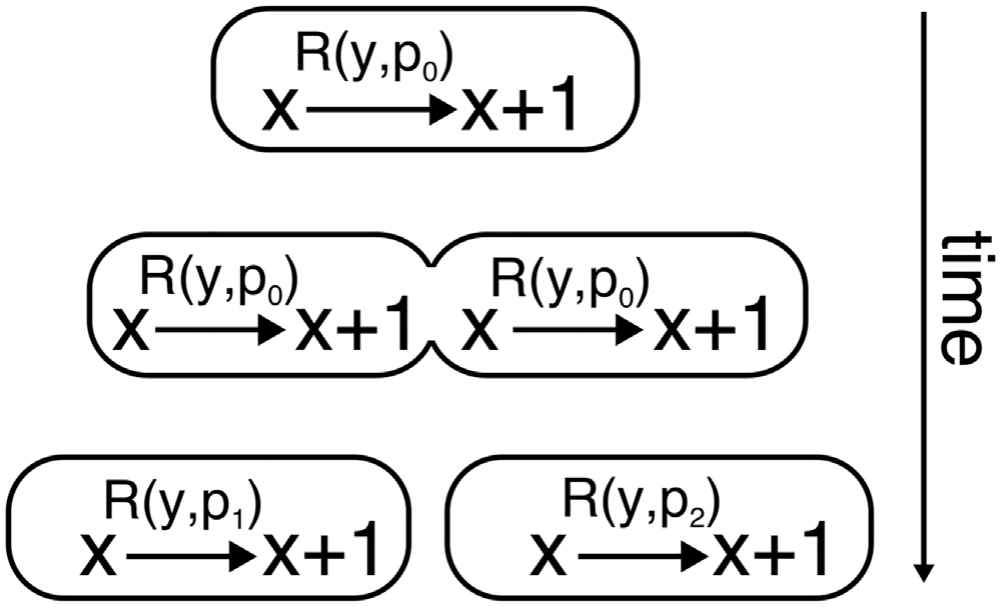
Implementation of parameter variability. A rate parameter *p* changing at cell division due to partitioning effects. While the cell is not dividing, *p* has the constant value *p*_0_ and the reaction rate is *R*(*y*, *p*_0_), where *y* is the state of the system. At cell division, the parameter *p* is different for each cell: *p*_1_ for cell 1, and *p*_2_ for cell 2. Cells 1 and 2 then have reaction rates *R*(*y*, *p*_1_) and *R*(*y*, *p*_2_). The value of *p* for the daugther cells will remain unchanged until the next division.

We implemented parameter variability in the model as described in Fig 13. For a reaction $x\overset{R(y,p)}{\to }x+1$ where *y* is the state of the system and *p* denotes a parameter of interest, the simulation proceeds as follows: When a cell is not dividing, the parameter has a constant value *p* = *p*_0_ and the reaction rate is $x\overset{R(y,p_{0})}{\to }x+1$. When the cell divides, the parameters for the sister cells may be different due to upstream fluctuations or partitioning effects. Thus, sister cell 1 is assigned the parameter *p* = *p*_1_ and cell 2 the parameter *p* = *p*_2_. After cell division the reaction rates are therefore different between the cells. Changes in the parameter *p* may represent transient changes in plasmid copy number or the availability of enzymes needed for protein production, as well as enzymes needed for protein degradation.

In our simulations for Figs 6 and 7 we modeled the evolution of a parameter of interest *p* by resampling it upon every division using the recursive relation

$$p^{i+1}∼N\left(qp^{i}+\left(1-q\right)\left\langle p\right\rangle ,\sigma_{p}^{2}\right).$$ (9)

Here *σ*_*p*_ was chosen so that the CV was Γ and 〈*p*〉 denotes the mean value of *p*. This is equivalent to *p* ^*i*+1^ = (*qp* ^*i*^ + (1 − *q*)〈*p*〉) (1 + Γ*η*), where η∼N(0,1). The parameter *q* represents the timescale of a homeostatic mechanism that ensures that the variance of *p* does not diverge. The initial parameters were themselves sampled from the stationary distribution of Eq (9), and parameters of daughter cells were sampled from a distribution determined by the parameters of the mother cell, $p_{\text{daughter}}∼N\left(qp_{\text{mother}}+\left(1-q\right)\langle p\rangle ,\sigma_{p}^{2}\right)$. The parameters of two sister cells, *p*_daughter_1__ and *p*_daughter_2__, were chosen so that their mean was equal to *p*_mother_ + (1 − *q*)*p*_mean_, that is (*p*_daughter_1__ + *p*_daughter_2__)/2 = *qp*_mother_ + (1 − *q*)*p*_mean_. For example, to model the evolution of the parameter that controls the production rate of the repressor, we initialized the system with a random value of $\alpha_{r}^{0}$, taken from the stationary distribution of the sequence $\alpha_{r}^{i+1}=\left(q\alpha_{r}^{i}+\left(1-q\right)\langle \alpha_{r}\rangle \right)\left(1+\Gamma \eta \right)$, where η∼N(0,1). Then, after one cell division, the new parameter was $\alpha_{r}^{1}=\left(q\alpha_{r}^{0}+\left(1-q\right)\langle \alpha_{r}\rangle \right)\left(1+\Gamma \eta \right)$. At the next division, we used $\alpha_{r}^{2}=\left(q\alpha_{r}^{1}+\left(1-q\right)\langle \alpha_{r}\rangle \right)\left(1+\Gamma \eta \right)$, and so on.

To account for variability in the copy number of plasmids, and enzymes required for protein production, we considered variability in the parameters *α*_*x*_ where *x* ∈ {*r*, *a*, *g*} and kept the others fixed. The mean value of the parameters were set to 〈*α*_*g*_〉 = 60*α*, 〈*α*_*a*_〉 = 60*α*/2, and 〈*α*_*r*_〉 = 25*α*/4, where *α* is given in Table 1. The parameters changed independently at cell division according to the rule $\alpha_{x}^{i+1}=\left(q\alpha_{x}^{i}+\left(1-q\right)\langle \alpha_{x}\rangle \right)\left(1+\Gamma \eta \right)$, for *x* ∈ {*r*, *a*, *g*}. To model the slow evolution of parameters we used *q* = *e* ^−ln(2)/5^, corresponding to a timescale of 5 cell generations. Fitting amplitude variability and cell-cell correlation yielded the values Ω = 2.1 and Γ = 0.12 (Table 3).

**Table 3:** Noise parameters. Parameters marked with * were estimated from Fig 6. These parameters with those from Tables 1 and 2 were used in Fig 7

**parameter**	**value**	**units**
*q*	exp(−ln(2)/5)	1
Ω*	2.1	1
Γ*	0.12	1

**Fig 14:**
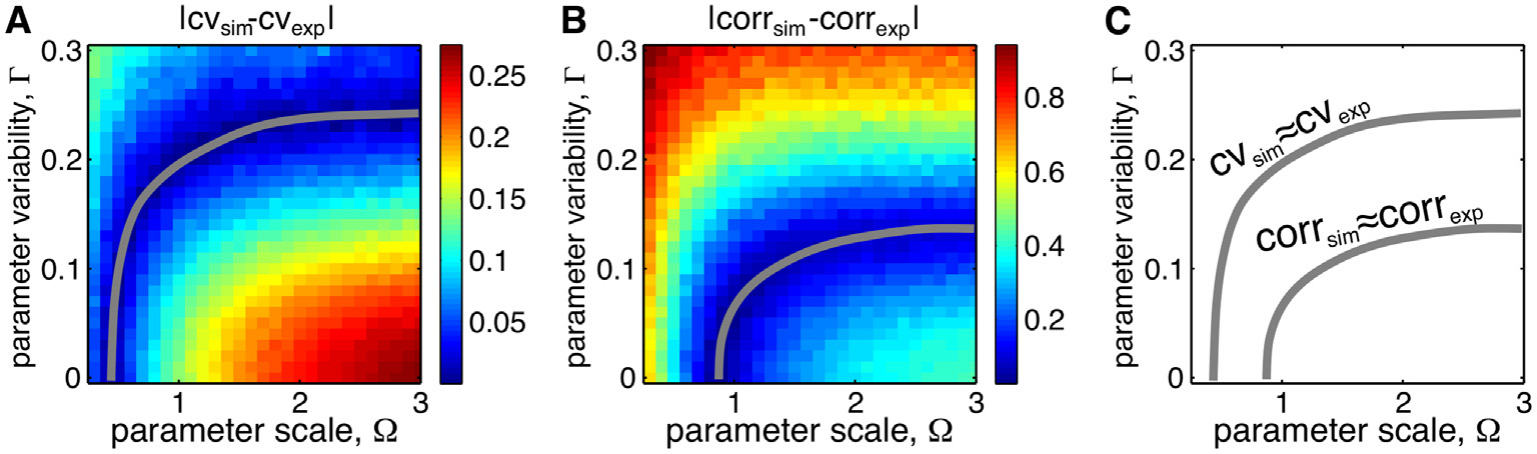
The effect of omitting lineage dependence. The parameter values at cell division are sampled from the same normal distribution regardless of the value of the parameters in the mother cell. **A.** Compared to the case of lineage dependence (Fig 6 in main text), the amplitude variability is lower and hence a higher level of parameter variability is needed to match the amplitude variability seen in experimental data. **B.** The amplitude correlation in simulations can match the experimental data by appropriately tuning the noise parameters Ω and Γ. **C.** The set of parameters obtained from panels A and B do not overlap, and it is not possible to match simultaneously the amplitude variability and correlation seen in experimental data.

We also considered simulations where the parameters were taken from the same distribution without changing the mean. That is, we considered $p_{\text{daughter}}∼N\left(\langle p\rangle ,\sigma_{p}^{2}\right)$, regardless of *p*_mother_. Note that this corresponds to using an instantaneous time scale of parameters, *q* = 0. With this choice, we could not find values of the parameters Ω and Γ that provided a match with experimental data (Fig 14). On the other hand, using $p_{\text{daughter}}∼N\left(p_{\text{mother}},\sigma_{p}^{2}\right)$, still allowed us to fit Ω and Γ. Note that this corresponds to using an infinitely slow time scale of parameters, *q* = 1. Thus, including the slow evolution of parameters was important in estimating the values of the noise sources.

### A toy oscillator model

Here we explore the effects of intrinsic and extrinsic noise on amplitude variability and correlation using the toy oscillator in Eq 6. We also illustrate that our method of estimating the levels of intrinsic and extrinsic noise can be expected to work in general.

The stochastic model in Eq 6 can be derived by transforming a 2 dimensional oscillator $\dot{x}=f\left(x,y\right)+\eta_{1}\left(t\right)$, $\dot{y}=g\left(x,y\right)+\eta_{2}\left(t\right)$ to polar coordinates, where *η*_1_(*t*) and *η*_2_(*t*) are independent noise terms [45]. Using *x* = *r*cos(*θ*), *y* = *r*sin(*θ*) we obtain

$$\begin{array}{cc} \dot{r} & =f\cos\left(\theta \right)+g\sin\left(\theta \right)+\eta_{1}\cos\left(\theta \right)+\eta_{2}\sin\left(\theta \right),\\ \dot{\theta } & =\frac{g\cos(\theta )-f\sin(\theta )}{r}+\frac{\eta_{2}\cos\left(\theta \right)-\eta_{1}\sin\left(\theta \right)}{r}. \end{array}$$

Since in steady state the periodic solution of this equation has constant radius, we can use its rotational symmetry. Then, *f*cos(*θ*) + *g*sin(*θ*) = *h*_1_(*r*) and *g*cos(*θ*) − *f*sin(*θ*) = *h*_2_(*r*). Also, we consider that *h*_1_(*r*) = *ρ*(*r*_0_ − *r*), where *ρ* again determines the stability of the limit cycle *r* = *r*_0_. If the period of the limit cycle is *T*, we can consider $h_{2}\left(r\right)=\frac{2\pi r_{0}}{T}$ (constant). We then obtain

$$\begin{array}{cc} \dot{r} & =\rho \left(r_{0}-r\right)+\eta_{1}\cos\left(\theta \right)+\eta_{2}\sin\left(\theta \right),\\ \dot{\theta } & =\frac{2\pi }{T}\frac{r_{0}}{r}+\frac{\eta_{2}\cos\left(\theta \right)-\eta_{1}\sin\left(\theta \right)}{r}. \end{array}$$

Because the system has rotational symmetry, we can consider

$$\begin{array}{cc} \dot{r} & =\rho \left(r_{0}-r\right)+\eta_{1}^{*},\\ \dot{\theta } & =\frac{2\pi }{T}\frac{r_{0}}{r}+\frac{\eta_{2}^{*}}{r}, \end{array}$$

where $\eta_{1}^{*}$ and $\eta_{2}^{*}$ are independent as in Eq 6. Parameter variability can then be incorporated as fluctuations in *r*_0_.

**Fig 15:**
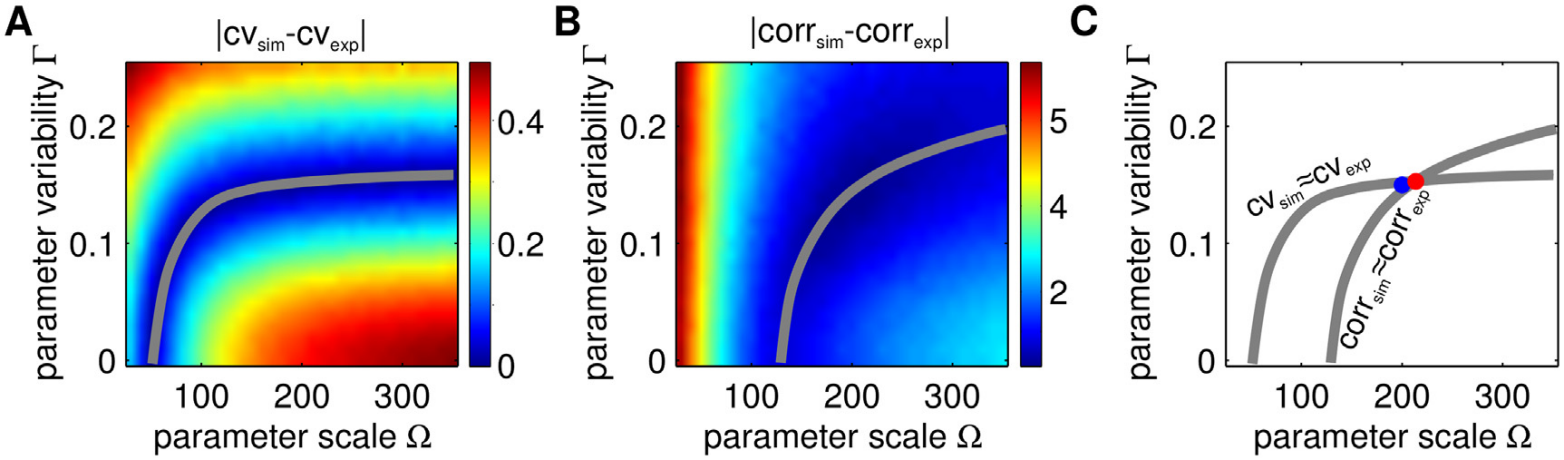
Testing our methodology on a toy model oscillator. We used *ρ* = 0.1min^−1^ (stability of the oscillator), *T* = 41min (period). The true levels of intrinsic and extrinsic noise chosen to generate *in silico* experimental data were Ω_0_ = 200 and Γ_0_ = 0.15 (blue dot in panel C). **A.** The difference between amplitude variability (gray curve shows the contour level that minimizes the difference). **B.** The difference between the amplitude correlation function (gray curve shows the contour level that minimizes the difference). **C.** The intersection of the curves of minima shown in panels A and B (red dot), provides an estimate of the true values of Ω and Γ (blue dot). The two curves intersect at (Ω_0_, Γ_0_) ≈ (200, 0.15) which was the original levels of noise to generate the *in silico* experimental data.

We tested if our method can obtain the true values of Ω and Γ using only the amplitude variability and correlation. First, we chose a fixed level of intrinsic and extrinsic noise: Ω_0_ = 200 and Γ = 0.15, and then generated simulations from which we computed the amplitude variability and the amplitude correlation: *var*_*exp*_ and *corr*_*exp*_ (*in silico* experimental data). Then, using the same analysis done for the full model, we computed the heatmaps of the difference between the amplitude variability from *in silico* experiments and from simulations (|*var*_*sim*_ − *var*_*exp*_|, Fig 15A) as well as the difference in amplitude correlation (|*corr*_*sim*_ − *corr*_*exp*_|, Fig 15B). Using these heatmaps, we found the predicted levels of noise to be (Ω, Γ) ≈ (200, 0.15), precisely the levels of noise used to generate the *in silico* experimental data (Fig 15C.) We also observe that intrinsic noise has a stronger effect on correlation than on amplitude variability, whereas extrinsic noise has a stronger effect on amplitude variability.

## Supporting Information

S1 Dataset Complete dataset. Spreadsheet containing data from each microscope run described in this article.
(XLSX)
